# Plasmid-Mediated Quinolone Resistance Genes in *Escherichia coli* Strains Isolated from Healthy Dogs

**DOI:** 10.3390/vetsci13030211

**Published:** 2026-02-25

**Authors:** Fatma Kalaycı-Yüksek, Defne Gümüş, Aysun Uyanık-Öcal, Aslı-Ceren Macunluoğlu, Mine Anğ-Küçüker

**Affiliations:** 1 Department of Medical Microbiology, Faculty of Medicine, Istanbul Yeni Yuzyil University, Istanbul 34010, Türkiye; defne.gumus@yeniyuzyil.edu.tr (D.G.); mine.kucuker@yeniyuzyil.edu.tr (M.A.-K.); 2 Department Medical Microbiology, Institute of Health Sciences, Istanbul University, Istanbul 34000, Türkiye; 3 Independent Researcher, Düzce 81000, Türkiye

**Keywords:** fecal *E. coli* healthy dogs, IncF and IncK plasmid replicon types, *qnr*-genes

## Abstract

Knowledge about the potential roles of pets as reservoirs for plasmid-mediated quinolone resistance is still limited in Türkiye. Thus, in our study, the presence of plasmid-mediated quinolone genes (*qnrA*, *qnrB* and *qnrS*) was examined by multiplex Polymerase Chain Reaction (PCR) in 101 fecal *Escherichia coli* (*Escherichia coli*) strains isolated from healthy dogs. Moreover, the relationship between the presence of *qnr* genes and prevalence of quinolone resistance, extended spectrum beta-lactamase (ESBL) and plasmid replicon types, mostly detected among fecal *E. coli* isolates (F, K, FIB, N, FIA, FIC, and Y) were investigated. A total of 41 strains (40.6%) carried at least one *qnr* gene. *Qnr* genes were found in 38.8% of quinolone-resistant and 40.9% of quinolone-susceptible strains. ESBL production was detected in 27 strains, 10 of which also harbored a *qnr* gene. Among *qnr*-positive strains, 19 (46.3%) carried both IncK and IncF plasmids (*p* < 0.001). IncF plasmids were significantly more prevalent in quinolone-resistant strains than in susceptible ones (*p* < 0.001), suggesting a potential link between *qnr* carriage, quinolone resistance, and IncF plasmids. To our knowledge, this is the first study investigating the relationship between *qnr* genes and specific plasmid replicon types in *E. coli* from healthy dogs in Türkiye. Our findings suggest that domestic animals may serve as reservoirs for antibiotic-resistant *E. coli*, underscoring the importance of a One Health approach.

## Simple Summary

Although it is well known that companion animals can serve as a source of zoonotic infectious diseases and antimicrobial-resistant bacteria for their owners, their potential role is often underestimated. To our knowledge, there are limited studies from Türkiye addressing the role of healthy companion animals in quinolone resistance. In the present study, the presence of plasmid-mediated quinolone genes, quinolone resistance, extended-spectrum beta-lactamase (ESBL) and plasmid replicon types, commonly found among fecal *Escherichia coli* isolates (F, K, FIB, N, FIA, FIC, and Y) were investigated. Among the 101 fecal *E. coli* strains examined, 41 (40.6%) were found to carry a *qnr* gene; of the 41 *qnr*-bearing strains, 19 (46.3%) harbored both IncK and IncF plasmid replicon types (*p* < 0.001). Our findings also suggest a possible association between *qnr* positivity, quinolone resistance, and IncF plasmid carriage. It may be concluded that companion animals may play a role in the dissemination of antibiotic-resistant *E. coli* strains.

## 1. Introduction

Resistant bacteria are an emerging problem all over the world. The overuse of antibiotics in human medicine, veterinary medicine and agriculture led to the domination of resistant bacterial populations [1,2,3,4,5]. It is known that not only clinical isolates, but also environmental isolates and members of the microbiota of humans, animals, and plants may serve as reservoirs for resistance genes [3,5,6,7,8,9,10]. One of the most important reservoirs may be the microbiota of domestic animals, particularly the intestinal microbiota, due to their close relationship with humans. At this point, the One Health concept represents an important approach that promotes collaboration among multiple science disciplines to protect health and take measures against antimicrobial resistance [11,12].

Quinolones are bactericidal antibiotics that block bacterial DNA replication via inhibition of DNA gyrase that catalyzes chromosomal DNA supercoiling [10,13]. Their extensive use in human and veterinary medicine has caused high resistance rates all over the world [14,15].

Although until the 1990s, it was believed that quinolone resistance could only occur by mutations of chromosomal genes coding for type II topoisomerases or efflux pumps and porins [13,16], plasmid-mediated quinolone resistance genes (*qnr*) were first defined in a *Klebsiella pneumoniae* strain by Martinez, Pascual and Jacoby in 1998 [17]. Since then, various types of *qnr* genes (*qnrA*, *qnrB*, *qnrS*, *qnrC*, *qnrD*, *qnrE* and *qnrVC*) were determined [13,16,18,19]. It is well known that plasmid-mediated resistance mechanisms have spread globally through the horizontal transfer of various resistance genes, such as extended-spectrum β-lactamases (ESBLs) and plasmid-mediated AmpC (pAmpC) enzymes [1,20,21,22,23]. In this context, bacterial conjugation represents a crucial mechanism facilitating the acquisition and dissemination of multidrug resistance [17,24,25].

Given the increasing global concern regarding antibiotic resistance, our previous study highlighted high rates of ESBL and quinolone resistance associated with close contact between humans and their companion animals [26]. Following these findings, the present study aimed to increase the number of isolates by focusing on canine samples and to determine the prevalence of plasmid-mediated quinolone resistance genes (*qnrA*, *qnrB*, and *qnrS*) in 101 *Escherichia coli* isolates. Furthermore, the distribution of plasmid replicon types commonly reported in our previous study and in the literature was investigated [23,24,25,26].

## 2. Materials and Methods

### 2.1. Strains

*Escherichia coli* strains were isolated from fecal samples of 101 healthy dogs (55 of which were included from our previous study), collected by their owners or veterinarians in Istanbul without any invasive procedures. All dogs included in the study were indoor pets, fed commercial dry food, and had no known history of antibiotic use within two weeks before sample collection. Fecal samples were plated on MacConkey and Chromogenic media and incubated at 37 °C for 24 h. Colonies showing typical *E.coli* morphology, based on lactose positivity on MacConkey agar and purple-pink colonies on chromogenic agar, were further identified by conventional biochemical tests (including oxidase, hydrogen sulfide and indole production, L-lysine decarboxylase activity, motility, glucose, sucrose and lactose fermentation, tryptophan deamination, and urea hydrolysis). After identification, bacteria were kept at −80 °C in tryptic soy broth (GBL, Istanbul, Türkiye) containing 20% glycerol for further analysis. This study was conducted from June 2019 to October 2025. Of the 101 *E. coli* strains included in the present study, 55 isolates originated from our previous study [26] and were collected between 2019 and 2022, while the remaining 46 strains were isolated between May 2023 and October 2025 and newly included in the present analysis.

### 2.2. Detection of Antibiotic Susceptibilities

The Kirby–Bauer disk diffusion method was used to determine the antibiotic susceptibilities of 101 *E. coli* strains. For this purpose, 17 different antimicrobial agents were used: ampicillin (AMP) (10 µg), cefotaxime (5 µg) (CTX), ceftazidime (5 µg) (CAZ), cefepime (30 µg) (FEP), cefoxitin (30 µg) (FOX), amoxicillin/clavulanate (AMC) (20/10 µg), piperacillin-tazobactam (30/6 µg) (TZP), imipenem (10 µg) (IMP), meropenem (10 µg) (MER), ertapenem (10 µg) (ERT), amikacin (10 µg) (AK), levofloxacin (5 µg) (LEV), ciprofloxacin (5 µg) (CIP), chloramphenicol (30 µg) (CL), gentamicin (10 µg) (GN), trimethoprim-sulfamethoxazole (1.25/23.75 µg) (SXT) and colistin (COL) (10 µg). The results were determined according to EUCAST Guidelines [27]. The double-disk synergy test was carried out using cefotaxime, ceftazidime and cefepime placed adjacent to amoxicillin-clavulanic acid to detect ESBL [28]. Colistin susceptibility was initially screened by the Kirby–Bauer disk diffusion method. Subsequently, carbapenem-resistant strains showing borderline or reduced susceptibility were further evaluated by the broth microdilution method using a commercial kit (Diagnostics s.r.o., Galanta, Slovakia), in accordance with CLSI guidelines [28]. We used *E. coli* ATCC 25922 as a control.

### 2.3. Detection of Plasmid-Mediated Quinolone Resistance Genes

The multiplex PCR method was used for the presence of plasmid-mediated quinolone genes (*qnrA*, *qnrB*, *qnrS*). Extracted (GeneDireX, Taoyuan City, Taiwan) and stored plasmids’ DNAs were analyzed as described previously [29]. The primers and PCR conditions used in our study are shown in Table 1. A commercial master mix kit (Genemark, Taichung City, Taiwan) was used for all PCR assays.

### 2.4. Detection of Plasmid Replicon Types

Plasmid replicon types (IncF, IncK, IncFIB, IncN, IncFIA, IncFIC, IncY replicons) commonly found in fecal *E.coli* strains isolated from dogs [23,24,25,26] were analyzed using two different multiplex PCRs [30,31] (Table 2). PCR conditions and primers are shown in Table 2.

### 2.5. Electrophoresis

All PCR products were visualized by electrophoresis (40 min, under 80 volts with 1XTBE) in 1.5% agarose gel stained by ethidium bromide (0.5 μg/mL). A DNA ladder (Genemark, Taichung City, Taiwan) ranging from 100 to 1000 bp was used.

### 2.6. Statistical Analysis

We evaluated all statistical associations using Pearson’s Chi-square test or Fisher’s exact test, as appropriate, and categorical variables are presented as *n* (%). Statistical analyses were performed using SPSS (IBM Corp. Released 2011. IBM SPSS Statistics for Windows, Version 20.0, Armonk, NY, USA: IBM Corp.), and a *p*-value < 0.05 was considered statistically significant.

## 3. Results

In this study, the numbers of strains resistant to the tested antibiotics were as follows: ampicillin 43 (42.6%), trimethoprim-sulfamethoxazole 31 (30.7%), cefotaxime 27 (26.7%), gentamicin 20 (19.8%), cefepime 18 (17.8%), ceftazidime 17 (16.8%), ciprofloxacin 17 (16.8%), levofloxacin 16 (15.8%), chloramphenicol 13 (12.9%), cefoxitin 9 (8.9%), amoxicillin/clavulanate 8 (7.9%), meropenem 4 (4%), piperacillin-tazobactam 4 (4%), ertapenem 4 (4%) and imipenem 3 (3%). No resistance was detected to amikacin and colistin. The presence of ESBL was detected in 27 (26.7%) of the isolates. Moreover, the number of resistance patterns associated with selected antibiotics of clinical importance was determined among the isolates, as follows: AMP + AMC: 8, CIP + LEV: 15, CTX + AMP: 24, GN + AMP: 14, and SXT + AMP: 28.

**Figure 1:**
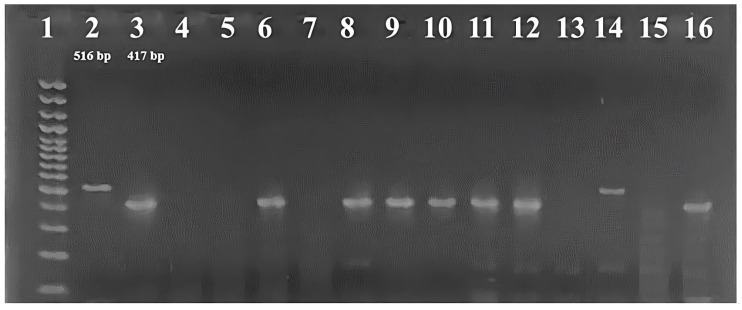
Agarose gel image of the *qnr* genes amplified by multiplex PCR. **well 1:** DNA Ladder (1000–100 bp); **well 2:** *qnrA* positive control; **well 3:** *qnrS* positive control; **well 4:** negative control; **well 5, 7, 13 and 15:** negative samples; **well 6, 8–12 and 16**: *qnrS* positive samples; **well 14:** *qnrA* positive sample.

Distributions of total *qnrA*, *qnrS*, and *qnrB* genes were 19 (18.8%), 17 (16.8%) and 5 (5%), respectively, among 101 fecal *E. coli* strains (Figure 1) (Table 3). In total, 41 (40.6%) of the 101 fecal *E. coli* strains were found to carry the *qnr* gene. None of the tested isolates harbored two or three *qnr* genes simultaneously. The detection rate of *qnr* was 38.8% in quinolone-resistant and 40.9% in quinolone-susceptible strains, and the difference was not statistically significant (*p* = 0.871). In quinolone-resistant isolates (18 strains), *qnrS* was the most frequently detected gene (4/7), whereas *qnrA* was the predominant gene among the 83 quinolone-susceptible isolates (17/34) (Table 3).

On the other hand, the prevalence of *qnr* was 10 (27%) among ESBL-positive strains, which is distributed as *qnrS*: 6 strain, *qnrA*: 3 strain and *qnrB*: 1 strain. There was no significant difference between ESBL-positive and ESBL-negative strains with respect to *qnr* gene presence/absence (*p*: 0.660) (Table 3).

The numbers of *qnr* genes in selected resistance patterns are also shown in Table 4. No statistically significant difference was observed between resistant and susceptible isolates in terms of the presence of total *qnr* genes for the combined resistance patterns AMP + AMC, CIP + LEV, AMP + CTX, GN + AMP, and SXT + AMP (*p* > 0.05).

In addition, both ESBL positivity and quinolone resistance were detected in 12 strains. Furthermore, three (25%) quinolone-resistant strains were identified as ESBL-positive and also carried the *qnr* gene. Among the ESBL-positive strains, no significant difference was observed between quinolone-resistant and quinolone-susceptible strains with respect to *qnr* gene positivity (*p*: 0.424). Carbapenemase positivity was detected in 4 strains (3.9%) in 101 fecal *E. coli* strains. One strain harboring the *qnr* gene was also found to be carbapenemase- and ESBL-positive.

The plasmid replicon types most frequently found in the intestinal microbiota (lncY, IncFIB, lncFlA, lncN, and IncK) were identified as IncY:11 (10.8%) strain, IncFlB: 24 (23.7%) strain, IncFIA: 10 (9.9%) strain, lncN:21 (20.8%) strain and IncK: 36 (35.6%) strain.

Furthermore, among the 41 *qnr*-bearing strains, 23 (56.1%) carried IncF or IncK plasmid replicon types, 12 (29.2%) carried IncN, and 3 (7.3%) carried lncY or lncFlA. Moreover, as a notable finding, 19 (47.3%) of the 41 *qnr*-bearing strains were found to carry both IncF and IncK replicon types. It was found that a significant difference between positive and negative strains for both IncF and IncK plasmids regarding *qnr* gene status (*p* < 0.001) was shown and *qnr* positivity was higher in the presence of IncF and IncK plasmids’ co-existence (Table 5).

Among the 18 Ievofloxacin- and/or ciprofloxacin-resistant strains, 15 (83.3%) harbored IncF, 6 (33.3%) harbored lncK, and 3 (16.7%) harbored the lncY plasmid replicon type (Table 5). In both ciprofloxacin- and Ievofloxacin-resistant strains, lncF positivity was significantly higher than quinolone susceptible strains (*p* < 0.001). No significant difference was detected in IncK and IncY positivity between resistant and susceptible strains for either antibiotic (Table 5). The complete raw data generated in this study are available as Supplementary Material in a separate Excel file [Supplementary Table S1 (Excel file)].

## 4. Discussion

Over the past century, the irrational usage of antibiotics in human and animal medicine has created selective pressure favoring resistant bacterial populations through horizontal gene transfer [8,32]. Moreover, several studies have reported the potential bidirectional transmission of resistant bacteria between animals and humans living in close contact [33,34,35,36]. In this context, previous studies suggest that pets’ frequent grooming behaviors and environmental interactions can contribute to contamination of their skin, mouth, and fur with fecal bacteria. Such bacteria may subsequently be transmitted to human cohabitants either directly through physical contact or indirectly via the shared household environment [1,37].

There are a limited number of studies investigating the prevalence of plasmid-mediated quinolone resistance among healthy pets in Türkiye to our knowledge [38,39,40]. Our results show that the prevalence of *qnr* genes was 40.6% in fecal *E. coli* strains obtained from healthy dogs, which is relatively higher than that reported in most previous studies conducted in companion animals. While lower prevalence rates have been reported in some studies [32,38,41,42,43,44], other studies detected no *qnr*-positive strains [23,45,46]. Our results are consistent with studies showing higher prevalence rates, such as de Jong et al., 2018 [10]. These results suggest that *qnr* genes may be frequently detected in the intestinal microbiota of healthy dogs in this region. These differences may be due to potential reasons such as geographic variation, differences in sampling, sample types and methodological approaches used in the respective studies.

On the other hand, *qnr* genes were also found in 34 strains (40.96%) among 83 quinolone-susceptible strains; similar detection rates have been reported in previous studies investigating quinolone-susceptible Enterobacterales strains where *qnr* genes were present despite phenotypic susceptibility [34,38,47,48,49,50]. This finding may be explained by the low-level expression of *qnr* genes, as previously described by Wang et al. [50].

Furthermore, the frequency of *qnr* genes was 37% in ESBL-positive strains, consistent with previous studies [38,42,44,49]. It has been demonstrated that healthy companion animals can harbor ESBL-producing and plasmid-mediated quinolone-resistant *E. coli* [38,42,44,49]. Although no significant difference was detected in *qnr* gene carriage between ESBL-positive and ESBL-negative strains, our findings support the notion that ESBL producers often exhibit co-resistance to quinolones and aminoglycosides. In addition to overall resistance rates, we identified strains exhibiting selected resistance patterns of clinical and epidemiological relevance. The detection rates of *qnr* genes in these selected patterns were 50%, 33.3%, 33.3%, 57.1 and 46.42 among strains showing resistance to AMP + AMC (8 strains), CIP + LEV (15 strains), CTX + AMP (24 strains), GN + AMP (14 strains), and SXT + AMP (28 strains), respectively. Monitoring such resistance patterns is important for understanding potential therapeutic challenges and the dissemination of multidrug-resistant *E. coli* in domestic animals. This resistance may result from the use of one of the two antibiotic classes, providing a co-selection mechanism [38,44,48,50,51]. Considering the role of plasmids in spreading ESBL and quinolone resistance, it is also possible that these resistance patterns are transferred via the same plasmids.

Most of the plasmid transfer of antimicrobial resistance genes provides a positive selection for certain resistance phenotypes among bacteria. Thus, it has been suggested that recognition and categorization of plasmids based on their phylogenetic relationships can be helpful both for the determination of their distributions in nature and their relationship with their host cells and for finding out their evolutionary origins [52,53].

Kalaycı-Yüksek et al. previously showed that the most detected plasmid replicon types were IncF (60%), IncK (58.1%), IncFIB (49%) and IncN (23.6%) in healthy dogs’ *E. coli* strains [26]. Consistent with these results, it has been shown that the IncF family is commonly found in the intestinal microbiota members of humans and animals independently from resistance genes [31,54]. On the other hand, the prevalence of other plasmid replicon types was shown to differ in previous studies, also showing their association with specific antimicrobial resistance genes [44,55,56,57].

In the present study, among the 41 *qnr*-encoding strains, the most prevalent plasmid replicon types were IncF (56.1%), IncK (56.1%) and IncN (29.2%). Moreover, as a noteworthy finding, we observed that 46.3% of these strains carried both IncF and IncK plasmids. This suggests that the most frequent plasmid replicon types may be co-transferred between strains. Therefore, it may also be possible to propose a relationship between the presence of the *qnr* gene and the carriage of IncF and IncK plasmids.

The carriage rate of the IncF replicon type was higher in strains resistant to both ciprofloxacin and levofloxacin than in quinolone-susceptible strains. This finding suggests that the observed quinolone resistance may also be caused by other plasmid-mediated resistance genes, such as *aac(6′)-Ib-cr* and efflux pump genes (e.g., *qepA*, *oqxAB*), which could be associated with the IncF plasmid [58,59] but were not examined in this study. Considering this finding, an association between the presence of the IncF plasmid type and quinolone resistance appears plausible. Taken together, these findings indicate that companion animals may represent a potential reservoir for antimicrobial resistance.

On the other hand, this study has several limitations. Feeding practices and antibiotic use were only known for up to two weeks before sampling, the dogs’ ages were not recorded, and other plasmid-mediated quinolone resistance genes were not examined. Future studies with larger sample sizes, including the owners, and including other *Enterobacterales* species, would enhance our understanding of plasmid-mediated quinolone resistance in the intestinal microbiota of healthy companion animals. Considering the close contact between humans and companion animals, such studies are essential to better understand potential consequences to public health.

## 5. Conclusions

The knowledge about the potential roles of pets as reservoirs for plasmid-mediated quinolone resistance is still limited in Türkiye. Therefore, in the present study, resistance to quinolones and the frequency of *qnr* genes were determined in fecal *E. coli* strains isolated from healthy dogs. This is the first study to report a high prevalence of *qnr* resistance genes in these isolates. Moreover, to our knowledge, the present study is the first to demonstrate an association between *qnr*-bearing fecal *E. coli* strains and IncF and/or IncK plasmid replicon types in Türkiye. Our results show that not only human isolates, but also animal and environmental strains could be responsible for the dissemination of resistance. Thus, today, the One Health approach is more important than ever.

## Figures and Tables

**Table 1:** Primers used for plasmid-mediated quinolone resistance by PCR analysis [29].

Genes	Primer Sequence (5′–3′)	Amplicon Size (bp)	PCR Condition
*qnrA F*	ATTTCTCACGCCAGGATTTG	516	Initial denaturation at 95 °C for 3 min, 30 cycles each consisting of 95 °C for 1 min, annealing temperature 55 °C or 30 s and 72 °C for 1 min, and a final extension step of 5 min at 72 °C
*qnrA R*	GATCGGCAAAGGTTAGGTCA		
*qnrB F*	GATCGTGAAAGCCAGAAAGG	469	
*qnrB R*	ACGATGCCTGGTAGTTGTCC		
*qnrS F*	ACGACATT CGTCAACTGCAA	417	
*qnrS R*	TAAATTGGCACCCTGTAGGC		

**Table 2:** Primers used for plasmid replicon types by multiplex PCR [30].

Name	Primer Sequence (5′–3′)	Amplicon Size (bp)	PCR Condition
**IncF F**	TGATCGTTTAAGGAATTTTG	270	Initial denaturation at 94 °C for 5 min was followed by 30 cycles of 94 °C for 1 min, annealing at 48 °C for 30 s, and extension at 72 °C for 1 min, with a final extension of 5 min at 72 °C.
**IncF R**	GAAGATCAGTCACACCATCC		
**IncK/B F**	GCGGTCCGGAAAGCCAGAAAAC	160	
**IncK R**	TCTTTCACGAGCCCGCCAAA		
**IncFIB F**	GGAGTTCTGACACACGATTTTCTG	702	
**IncFIB R**	CTCCCGTCGCTTCAGGGCATT		
**IncN F**	GTCTAACGAGCTTACCGAAG	559	
**IncN R**	GTTTCAACTCTGCCAAGTTC		
**IncFIA F**	CCATGCTGGTTCTAGAGAAGGTG	462	Initial denaturation at 94 °C for 5 min was followed by 30 cycles of 94 °C for 1 min, annealing at 59 °C for 30 s, and extension at 72 °C for 1 min, with a final extension of 5 min at 72 °C.
**IncFIA R**	GTATATCCTTACTGGCTTCCGCAG		
**IncFIC F**	GTGAACTGGCAGATGAGGAAGG	262	
**IncFIC R**	TTCTCCTCGTCGCCAAACTAGAT		
**IncY F**	AATTCAAACAACACTGTGCAGCCTG	765	
**IncY R**	GCGAGAATGGACGATTACAAAACTTT		

**Table 3:** The presence of *qnr* genes, quinolone susceptibilities and ESBL production in *E. coli* strains.

*qnr* Genes	Quinolone-Susceptible		Quinolone-Resistant		ESBL-Positive		ESBL-Negative	
***n* = 41**	***n* = 83**	**%**	***n* = 18**	**%**	***n* = 27**	**%**	***n* = 74**	**%**
***qnrA***	17	20.5	2	11.1	3	11.1	16	21.6
***qnrB***	4	4.8	1	5.6	1	3.7	4	5.4
***qnrS***	13	15.7	4	22.2	6	22.2	11	14.9

**Table 4:** Prevalence of *qnr* genes in *E. coli* strains with selected resistance patterns.

*qnr* Genes (*n* = 41)	AMC + AMP (*n* = 8)	CIP + LEV (*n* = 15)	CTX + AMP (*n* = 24)	GN + AMP (*n* = 14)	SXT + AMP (*n* = 28)
***qnrA***	1	1	3	3	5
***qnrB***	1	1	1	2	1
***qnrS***	2	3	5	3	7

**Table 5:** The plasmid replicon types in quinolone-susceptible/resistant strains.

Plasmid Replicon Types		Quinolone-Susceptible (*n* = 83)	Quinolone-Resistant (*n* = 18)	*p*-Value
IncF	Negative	63 (75.9%)	3 (16.7%)	**<0.001 ^a^**
	Positive	20 (24.1%)	15 (83.3%)	
IncK	Negative	53 (63.9%)	12 (66.7%)	0.821 ^a^
	Positive	30 (36.1%)	6 (33.3%)	
IncY	Negative	75 (90.4%)	15 (83.3%)	0.408 ^b^
	Positive	8 (9.6%)	3 (16.7%)	

a: Pearson chi-square test, b: Fisher’s exact test.

## Data Availability

The original contributions presented in this study are included in the article. Further inquiries can be directed to the corresponding author.

## Supplementary Materials

The following supporting information can be downloaded at: https://www.mdpi.com/article/10.3390/vetsci13030211/s1, Table S1: Distribution of antibiotic resistance profiles, ESBL and carbapenemase production, *qnr* positivity and plasmid replicon types among the examined isolates.
