# Supplementary material for: Plasmid-Mediated Quinolone Resistance Genes in Escherichia coli Strains Isolated from Healthy Dogs
Source: Vet Sci. 2026 Feb 25;13(3):211. doi: 10.3390/vetsci13030211 (PMC13029972; doi:10.3390/vetsci13030211)
Supplement: Supplementary file 1 [file vetsci-13-00211-s001.zip › vetsci-4097838,figure S1-original_images.zip!/vetsci-4097838,figure S1-original_images.pdf]

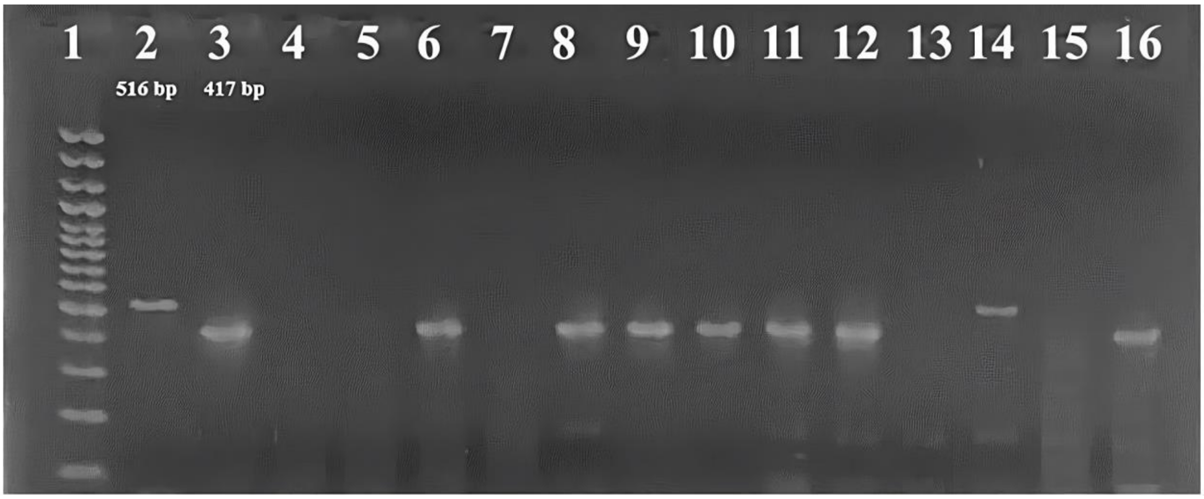

Figure S1: Western blot original figure.
